# Circulating Adipokines in Healthy versus Unhealthy Overweight and Obese Subjects

**DOI:** 10.1155/2014/170434

**Published:** 2014-01-16

**Authors:** Assim A. Alfadda

**Affiliations:** Obesity Research Center and the Department of Medicine, College of Medicine, King Saud University, P.O. Box 2925 (98), Riyadh 11461, Saudi Arabia

## Abstract

It is now well established that not all obese subjects are at increased risk of cardiometabolic complications; such patients are termed the metabolically healthy obese. Despite their higher-than-normal body fat mass, they are still insulin sensitive, with a favorable inflammatory and lipid profile and no signs of hypertension. It remains unclear which factors determine an individual's metabolic health. Adipose tissue is known to secrete multiple bioactive substances, called adipokines, that can contribute to the development of obesity-associated complications. The goal of this study was to determine whether the circulating adipokine profiles differs between metabolically healthy and metabolically unhealthy overweight and obese subjects, thereby obtaining data that could help to explain the link between obesity and its related cardiometabolic complications. We defined metabolic health in terms of several metabolic and inflammatory risk factors. The serum adiponectin levels were higher in the healthy group and showed a positive correlation with HDL cholesterol levels in the unhealthy group. There were no differences between the two groups in the levels of serum leptin, chemerin and orosomucoid. Accordingly, adiponectin might play a role in protecting against obesity-associated cardiometabolic derangements. More studies are needed to clarify the role of different chemerin isoforms in this system.

## 1. Introduction

The prevalence of obesity is increasing worldwide, accompanied by a high incidence of type 2 diabetes (T2DM) and cardiovascular disease (CVD) [1]. Although there is convincing evidence that obesity is accompanied by unfavorable metabolic profiles, such as impaired glucose tolerance, dyslipidemia, elevated blood pressure, or low-grade systemic inflammation, this may not always be the case. Some obese individuals do not possess this constellation of metabolic abnormalities and have been termed the metabolically healthy (MH) obese [2, 3]. Several studies have shown that MH obese participants are not at an increased risk of developing CVD compared with healthy, normal-weight participants [4–7]; certainly, they are at lower risk than metabolically unhealthy obese participants [8]. However, these results are in contrast to the data from several studies reporting that excess weight, as determined by body mass index (BMI), was associated with the incidence of CVD, even after adjusting for traditional metabolic risk factors [9, 10]. Hence, it is likely that there is a direct effect of excess weight on the risk of CVD as a result of complex biological mechanisms other than blood pressure, diabetes, and cholesterol.

It is now well known that adipose tissue functions as an endocrine organ that secretes various bioactive molecules (called adipokines), that possess pro- and anti-inflammatory activities. In obesity, adipose tissue expansion causes adipocyte dysfunction and an imbalance in the secretion of these molecules, which has been linked to the initiation and progression of obesity-associated complications [11]. Furthermore, a state of low-grade inflammation in obese individuals has been associated with increased circulating levels of the proinflammatory marker C-reactive protein (CRP), which has been reported to predict the development of T2DM in various populations [12–14]. Only a few studies have examined the adipokine profiles of MH obese and overweight subjects [15–17]. Furthermore, no data are available on recently discovered adipokines, for example, chemerin and orosomucoid, and there are no published studies on the adipokine profiles in MH obese and overweight Arabs.

To further understand the complex relationship between excess body fat and the development of obesity-associated metabolic and cardiovascular complications, we sought to study the circulatory profiles of several adipokines in a well-characterized group of overweight and obese individuals. For the MH group, the clinical and biochemical data did not show any evidence of metabolic derangements or inflammation, whereas for the non-MH signs of metabolic and inflammatory derangements had already begun to develop. The circulating levels of leptin, adiponectin, chemerin and orosomucoid were reported in these two groups of overweight and obese subjects and then correlated with several metabolic and inflammatory markers. Our goal was to determine whether the circulating adipokine profiles differed between the MH group and the non-MH group, which may help to explain the unhealthy metabolic profiles of the non-MH group, despite these individuals having the same degree of adiposity as the MH individuals.

## 2. Materials and Methods

The subjects were individuals undergoing elective abdominal surgery for cholecystectomy and weight reduction. Eighty nine consecutive subjects (fifty seven females) were recruited. The subjects' ages ranged from 17 to 70 years, and were either overweight (BMI ≥ 25 kg/m^2^) or obese (BMI ≥ 30 kg/m^2^). All subjects had a stable weight with no fluctuations >2% of their body weight for at least 2 months prior to study. The exclusion criteria included the presence of acute inflammation, infection, or malignant conditions. This study was conducted at the Obesity Research Center, College of Medicine, King Saud University, Riyadh, Saudi Arabia. The Institutional Review Board approved this study, and all participants gave informed consent.

Weight (in kilograms) was measured in light clothing, without shoes, to the nearest 0.1 kg; height was measured using a stadiometer to the nearest centimeter; and BMI was calculated (weight/height squared; in kilograms per square meter). BMI ranged from 25.1 to 66.8 kg/m^2^. The percentage of body fat was measured by a bioelectrical impedance analyzer system (BT-905 Body Composition Analyzer, Skylark Co. Ltd., Taoyuan, Taiwan). After overnight fasting, blood samples were obtained, and sera were stored at −80°C, until analytical measurements were performed.

The levels of serum glucose, triglycerides, total cholesterol, and high-density lipoprotein (HDL) cholesterol were determined using a Dimension Xpand Plus integrated clinical chemistry autoanalyzer (Siemens Healthcare Diagnostics, Deerfield, IL, USA). The serum levels of low-density lipoprotein (LDL) cholesterol were calculated using Friedewald's equation [18]. The plasma insulin quantity was determined by electrochemiluminescence using a Cobas e411 immunoanalyzer (Roche Diagnostics, Indianapolis, IN, USA). Insulin resistance was represented using the “homeostasis model assessment of insulin resistance” (HOMA-IR), which was determined according to the following equation: HOMA-IR = fasting plasma glucose (mmol/L) × fasting plasma insulin (mIU/L)/22.5 [19]. Glycosylated hemoglobin (HbA1c) was measured using a turbidimetric inhibition immunoassay with the Dimension Xpand Plus autoanalyzer (Siemens Healthcare Diagnostics).

Commercially available ELISA kits were used to measure the serum concentrations of leptin, adiponectin, chemerin (Millipore Corporation, Billerica, MA, USA), orosomucoid (AssayMax Human *α*-1-Acid Glycoprotein, AssayPro, Saint Charles, MO, USA), and CRP (Immundiagnostik AG, Bensheim, Germany) according to the manufacturers' recommended protocols (leptin intra-assay CV = 4.9% and inter-assay CV = 8.6%; adiponectin intra-assay CV = 7.4% and inter-assay CV = 8.4%; chemerin intra-assay CV 5.0% and inter-assay CV 6.0%; orosomucoid intra-assay CV 4.4% and inter-assay CV 7.2%; and CRP intra-assay CV = 6.0% and inter-assay CV = 11.6%).

Metabolic risk was based on an adaptation of the previous criteria [6, 20, 21]. Subjects were classified as metabolically healthy if they had fewer than two components. The components are the following: systolic/diastolic blood pressure ≥130/85 mmHg or antihypertensive medication use; fasting triglycerides level ≥1.7 mmol/L; HDL-cholesterol level <1.04 mmol/L in men or <1.3 mmol/L in women or lipid-lowering medication use; fasting glucose level ≥5.56 mmol/L or antidiabetic medications use; HOMA-IR >75th percentile; hsCRP level >3 mg/L.

*Statistical Analysis.* Statistical analysis was performed using SPSS version 17 (Chicago, Illinois, USA). Before the statistical analysis, a logarithmic transformation of the nonnormally distributed parameters was performed to approximate a normal distribution. Data are shown as the means ± SD unless stated otherwise. The comparison of the clinical and biochemical characteristics between the MH and non-MH groups was performed using student's *t*-test for independent samples and box plots. Correlations (*r*) between variables were quantified by Karl Pearson's correlation analysis. Multiple linear regression analysis was performed to test the independent linear relationship between the serum adipokine levels and the markers of glycemia, insulin resistance, lipids and inflammation, after adjusting for the effects of age, sex, and BMI. Scatter diagrams with regression lines were used to show the linear relationships of these quantitative variables. A *P* value of ≤0.05 was considered statistically significant.

## 3. Results

The characteristics of the overall study population are listed in Table 1. All of the studied patients were either overweight (BMI ≥ 25 and <30 kg/m^2^, *n* = 12) or obese (BMI ≥ 30 kg/m^2^, *n* = 77). The same table shows the characteristics of the MH and the non-MH subjects. There were no differences between the groups in relation to age, BMI, % body fat, or blood pressure. The non-MH group was more insulin-resistant, with a higher fasting glucose level, a higher serum triglyceride level, a lower HDL cholesterol level, and a higher CRP level.

**Figure 1:**
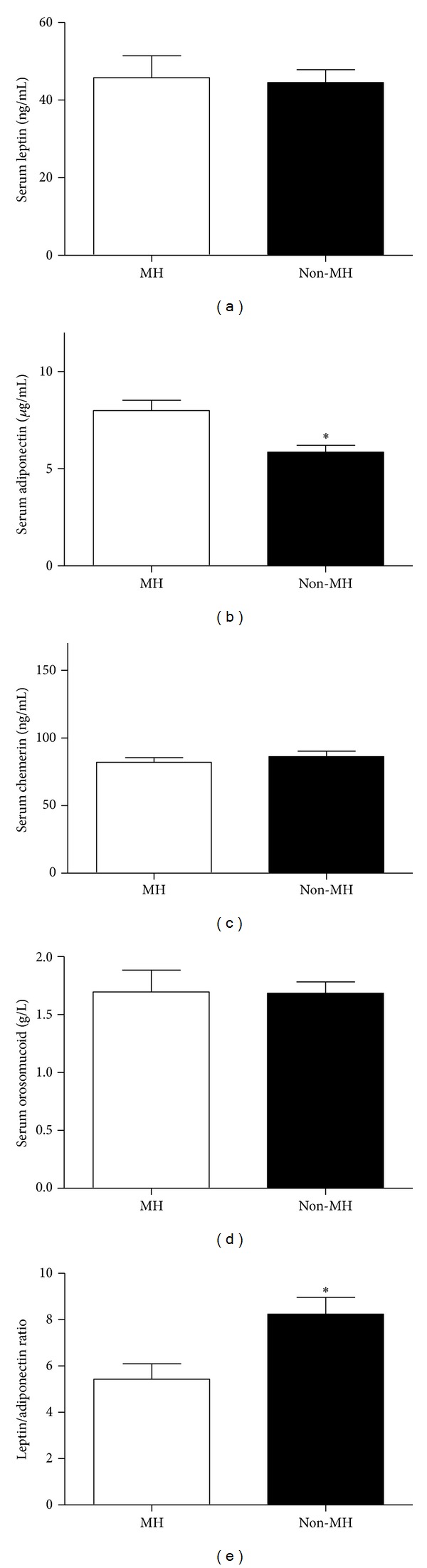
Serum leptin, adiponectin, chemerin, and orosomucoid in metabolically healthy and unhealthy subjects. No significant difference in the levels of serum leptin, chemerin, or orosomucoid was found between the two groups. Serum adiponectin level was significantly lower in the metabolically unhealthy group (*P* < 0.001). The leptin to adiponectin ratio was significantly higher in the metabolically unhealthy group (*P* < 0.01).

Serum adiponectin levels were significantly higher in the MH subjects (8 ± 3 versus 5.9 ± 2.6 *μ*g/mL in the non-MH, *P* < 0.001) than the non-MH subjects. There were no differences between the two groups in terms of the levels of serum leptin (45.8 ± 27.8 in MH versus 44.5 ± 21.1 ng/mL in non-MH, *P* = 0.8), chemerin (82 ± 18 in MH versus 86 ± 30.1 ng/mL in non-MH, *P* = 0.5), or orosomucoid (1.7 ± 0.8 in MH versus 1.7 ± 0.6 g/L in non-MH, *P* = 0.94). The leptin to adiponectin ratio was lower in the MH subjects (5.4 ± 3.2 versus 8.2 ± 4.6 in the non-MH, *P* < 0.01) (Figure 1).

**Figure 2:**
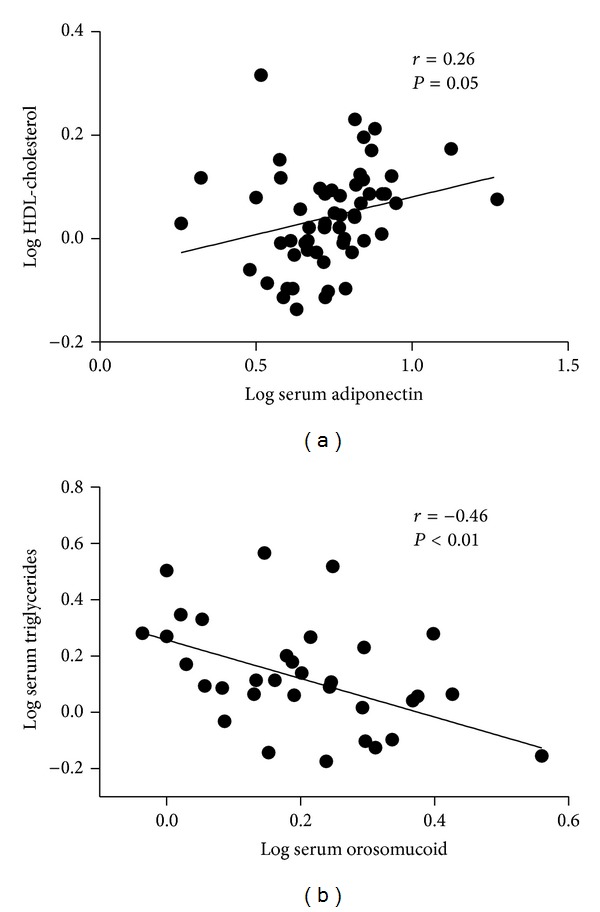
The correlation of serum adipokine levels with different metabolic risk components. Serum adiponectin level was significantly and positively correlated with HDL cholesterol (*r* = 0.26, *P* = 0.05) (a). Serum orosomucoid level were significantly and negatively correlated with serum triglycerides level (*r* = −0.46, *P* < 0.01) (b).

We performed a correlation analysis to investigate whether circulating adipokine levels were related to the metabolic and inflammatory risk components in the non-MH group, including the following: fasting plasma glucose, HOMA-IR, lipid profile, and CRP. There was a significant positive correlation between the serum leptin level and LDL cholesterol (*r* = 0.33, *P* = 0.03), as well as a significant negative correlation between serum leptin and serum triglycerides (*r* = −0.46, *P* < 0.01). The serum adiponectin level was negatively correlated with HOMA-IR (*r* = −0.31, *P* = 0.02) and positively correlated with HDL cholesterol (*r* = 0.26, *P* = 0.05) (Figure 2(a)). The serum orosomucoid level was negatively correlated with serum triglycerides (*r* = −0.46, *P* < 0.01) (Figure 2(b)). There was no significant correlation between serum chemerin and any of the metabolic or inflammatory risk components.

After adjusting for age, sex, and BMI, the above correlations remained significant except the correlation between serum leptin level and LDL cholesterol level (*P* = 0.88), serum leptin level and serum triglycerides level (*P* = 0.1), and serum adiponectin level and HOMA-IR (*P* = 0.47) which became non-significant.

## 4. Discussion

A considerable proportion of overweight and obese adults are metabolically healthy. Although their phenotype shows an excess of body fat, they do not have metabolic abnormalities, such as insulin resistance, dyslipidemia, hypertension, or unfavorable inflammatory profiles [20, 22]. Several definitions have been proposed to define metabolic health, leading to widely varying estimates of the prevalence of metabolically healthy overweight and obese individuals depending on which definition is used. In a cross-sectional sample of 5440 participants from the National Health and Nutrition Examination Surveys (NHANES 1999–2004), Wildman et al. reported that 51.3% of overweight and 31.7% of obese adults were metabolically healthy [20]. Others have reported a wide range of prevalences, from 6% to 40% in different overweight and obese populations [6, 22–24]. In the present study, 37% of the subjects were MH, which is considered amongst the highest reported prevalence. We believe that the primary cause of this variability is related to differences in how metabolic health is defined. Study design differences, such as age group, sample size, and ethnicity, could also contribute to this disparity.

It is now well known that adipose tissue is not simply a reservoir for fat storage, but rather, adipose tissues constitute an active endocrine organ with multiple roles. One of these important roles is the contribution of adipose tissue to the inflammatory process in both vascular and nonvascular tissues [25]. Activated macrophages, together with adipocytes, secrete a number of proteins (called adipokines) that have both pro- and anti-inflammatory activities. Adipocyte dysfunction occurs as a result of excess fat accumulation in the body, which causes dysregulated production of adipokines, thereby contributing to the pathogenesis of obesity-associated metabolic and cardiovascular complications. The study of adipokine profiles, therefore, is central to understanding why some individuals develop obesity-associated complications, and others do not. Thus, for a cohort of MH and non-MH overweight and obese individuals, we reported the levels of leptin, an adipokine that regulates feeding behavior through the central nervous system and acts as a proinflammatory adipokine; adiponectin, an anti-inflammatory adipokine that plays a role in protection against the development of metabolic and cardiovascular complications related to obesity; chemerin, which plays several roles in both inflammation and metabolism and has been proposed as a link between chronic inflammation, obesity, and obesity-related comorbidities; and orosomucoid, which is proposed to modulate the immune responses to protect adipose tissues from the effects of excessive inflammation and metabolic dysfunction [11, 26, 27].

We found that serum adiponectin was significantly higher in the MH subjects than non-MH subjects. This confirms the results of other studies [23, 28]. In our study, we noticed that higher adiponectin levels were associated with less insulin resistance and higher HDL cholesterol. This was still the case after correcting for body weight. This further confirms that overweight and obese individuals with higher adiponectin levels are more likely to show a favorable metabolic and inflammatory profile and hence less likely to develop cardiovascular and metabolic complications. Adiponectin has anti-inflammatory and vascular protective effects, which makes it an attractive therapeutic target. Indeed, several pharmacological approaches are currently under extensive evaluation, including the following: (i) agents that augment adiponectin circulating concentrations, (ii) agents that increase adiponectin receptor expression, and (iii) agonists of the adiponectin receptor, which induce signaling pathways downstream of adiponectin receptors [29].

Although it has been shown previously that serum leptin correlates positively with BMI and % body fat [11], its level in the MH group was similar to that in the non-MH group. Supporting this finding, Koster et al. did not find significant difference in the serum leptin levels between MH and non-MH subjects in a cohort of obese older adults [15]. Because these two adipokines, namely, leptin and adiponectin, are closely linked to fat metabolism and are considered as an important mediators linking adiposity to atherosclerosis [30], we calculated the leptin to adiponectin ratio in both groups. We report here that the leptin to adiponectin ratio was lower in the MH subjects compared to the non-MH. It has been proposed that this ratio could be utilized as a surrogate marker to assess metabolic syndrome and atherosclerosis in obese type 2 diabetic patients [31, 32]. We found no previous reports on this ratio in MH obese, nondiabetic individuals. Therefore, it will be important to assess whether applying measures that decrease this ratio would help in preventing, or at least in decreasing, the progression of atherosclerosis in obese non-diabetic individuals.

Previous studies have confirmed that serum chemerin and orosomucoid correlate positively with BMI and % body fat [33, 34]. From the few studies that looked at the adipokine levels in MH, none reported chemerin or orosomucoid levels. We showed here that these levels in MH subjects were similar to those in non-MH individuals. Although several groups have reported a proinflammatory role for chemerin, some studies have linked chemerin expression to anti-inflammatory actions [35]. This could be explained by the presence of multiple chemerin-derived peptides with different inflammatory roles. In the present study, we measured the total serum chemerin which could explain our failure to detect a difference in the circulating level of chemerin between the two groups. Studying the role of different chemerin-derived peptides in overweight and obese individuals could give different results.

It has been shown that orosomucoid secretion from the adipocytes was markedly increased by inflammatory and metabolic stress mediators, including TNF-*α*, insulin, glucose and free fatty acids [34]. This finding, then, may constrain excess inflammatory response in adipose tissues by suppressing the interactions between adipocytes and macrophages. We previously reported that circulating orosomucoid correlate positively with BMI and body fat mass [27]. In the present study, we did not find a significant difference in the levels of circulating orosomucoid between MH and non-MH subjects. However, the serum orosomucoid levels in our cohort were significantly higher than those reported under normal physiological conditions [36]. It is possible, therefore, that the body fat mass, rather than the metabolic status of the subject, primarily determines the circulatory level of orosomucoid.

Our findings are limited by the small sample size. Larger studies are therefore required to confirm our findings. Furthermore, we reported the total chemerin levels, even though the precise level of bioactive chemerin, its relation to obesity and its associated comorbidities remain to be examined. Despite these limitations, our study has a number of strengths. It is the first to report the levels of adipokines in the MH overweight and obese Arab population. Several previous studies have defined metabolic health based on definitions of metabolic syndrome or on an insulin resistance cut-off point alone. The present study included several metabolic syndrome components, in addition to the insulin resistance and inflammation criteria, and thus addressed a wider range of metabolic abnormalities.

In conclusion, we have reported the circulatory profiles of several adipokines in a well-characterized group of MH and non-MH obese and overweight individuals. The findings of this study contribute towards a better understanding of the imbalance between pro- and anti-inflammatory adipokines and of obesity-induced metabolic comorbidities, which could aid in the design of better preventive and therapeutic strategies against this disease.

## Figures and Tables

**Table 1:** Clinical and biochemical characteristics of the study population.

Variable	All	MH	non-MH	*P*
Number (*n*)	89	33	56	
Age (years)	36.6 ± 12.5	34.8 ± 11.3	37.7 ± 13.1	0.29
BMI (kg/m^2^)	42.6 ± 10.2	41.1 ± 10.6	43.6 ± 10	0.26
Body fat (%)	51.2 ± 13	49.2 ± 15.3	52.3 ± 11.4	0.3
SBP (mmHg)	123.6 ± 11.8	120.8 ± 11.9	125.6 ± 11.5	0.09
DBP (mmHg)	70.8 ± 8	70.4 ± 8	71.1 ± 8.1	0.69
Fasting glucose (mmol/L)	6 ± 1.78	5.3 ± 0.8	6.4 ± 2	**0.00**
Insulin (mIU/L)	15.8 ± 9.8	11.1 ± 4.2	18.4 ± 11.1	**0.00**
HOMA-IR	4.3 ± 3.5	2.6 ± 1.1	5.2 ± 4.1	**0.00**
HbA1c	5.9 ± 1	5.6 ± 0.4	6.1 ± 1.2	0.09
Total cholesterol (mmol/L)	4.9 ± 1	4.8 ± 0.8	5 ± 1	0.6
LDL cholesterol (mmol/L)	3 ± 0.8	2.9 ± 0.7	3.1 ± 0.8	0.3
HDL cholesterol (mmol/L)	1.2 ± 0.3	1.3 ± 0.2	1.1 ± 0.3	**0.00**
Triglycerides (mmol/L)	1.4 ± 0.7	1.1 ± 0.3	1.5 ± 0.8	**0.00**
HsCRP (mg/L)	12 ± 11.2	7.1 ± 6.3	14.2 ± 12.2	**0.01**

Data are presented as the means ± standard deviation. BMI: body mass index; SBP: systolic blood pressure; DBP: diastolic blood pressure; LDL: low-density lipoprotein; HDL: high-density lipoprotein. The *P* values were calculated from the MH versus non-MH comparison of the values for each of the measured parameters. Values <0.05 (bolded) were considered statistically significant.
